# Ureteric Stone Management in Crossed-Fused Renal Ectopia With Bilateral Duplex-Collecting System

**DOI:** 10.1155/2024/2662107

**Published:** 2024-10-10

**Authors:** Abdoulhafid Elmogassabi, Tawiz Gul, Bela Tallai, Maged Alrayashi, Mohamed Abdelkareem, Mohammed Ibrahim, Abu Baker, Mohammed Ebrahim, Hossameldin Alnawasra, Salvan Alhabash, Morshed Salah

**Affiliations:** ^1^Urology Section, Surgery Department, Hazm Mebaireek General Hospital, Hamad Medical Corporation, Doha, Qatar; ^2^College of Medicine, Qatar University, Doha, Qatar

**Keywords:** crossed-fused renal ectopia, duplex system, ureteric stone

## Abstract

Crossed-fused renal ectopia (CFRE) is a rare congenital anomaly where both kidneys are fused on the same side. We report a case of a 52-year-old male patient who presented with central abdominal pain associated with hematuria and dysuria, with a history of left open ureterolithotomy. Abdominal computed tomography (CT) showed an 18-mm left distal ureteric stone and a CFRE with a bilateral duplex-collecting system. Left ureteroscopy and laser lithotripsy with magnetic double-J stent insertion were carried out successfully, and the patient was discharged on the same day in fair general condition.

## 1. Introduction

Crossed-fused renal ectopia (CFRE) is a rare congenital anomaly in which the kidneys are located on the same side. Its frequency is around 1/7000 [1]. Most cases are asymptomatic and are diagnosed incidentally [1, 2]. The anomaly predisposes patients to urinary obstruction, infection, neoplasia of the urinary system, and nephrolithiasis [3]. CFRE usually does not require any primary treatment unless complications occur [4].

## 2. Case Presentation

**Figure 1:**
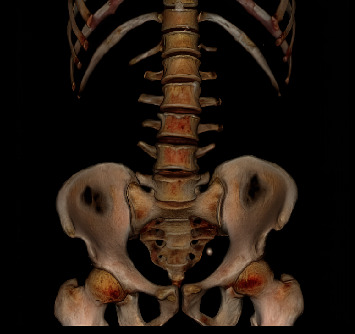
Three-dimensional CT reconstruction shows an 18-mm left lower ureteric stone.

**Figure 2:**
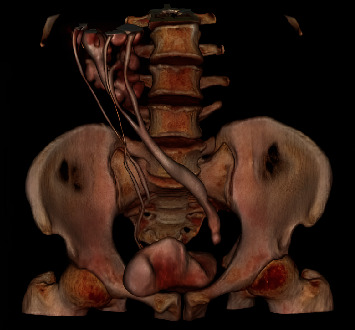
Three-dimensional CT reconstruction showed a crossed-fused ectopia, where the left kidney is fused to the lower renal pole of the right kidney.

**Figure 3:**
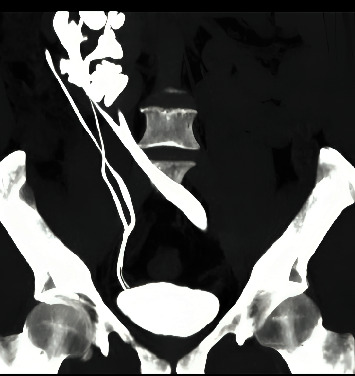
CT urography showed that the right kidney had a complete duplex system with two separate ureters, while an incomplete duplex system was found on the left side.

**Figure 4:**
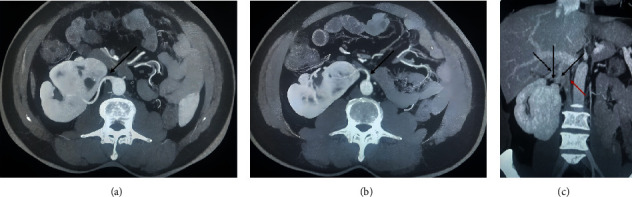
(a–c) The right side crossed renal ectopia supplied by two renal arteries. Both renal arteries arise below the level of the inferior mesenteric artery and are drained by multiple renal veins to the IVC.

**Figure 5:**
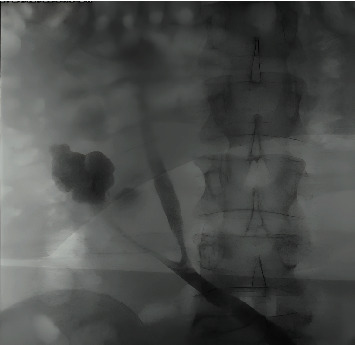
Left retrograde pyelography (RGP) showed moderate hydroureteronephrosis and duplex ureter uniting proximally.

A 52-year-old male patient presented with a history of central abdominal pain for 3 days, radiating to the groin, associated with hematuria and dysuria. He had a history of stone disease and underwent left open ureterolithotomy many years ago. Physical examination revealed a left iliac fossa scar. The urine culture was negative, and serum creatinine (Cr) was normal. A contrast-enhanced computed tomography (CT) with three-dimensional reconstruction showed a variant CFRE, where the left kidney was found to be fused to the lower pole of the right one. The right kidney had a complete duplex system with two separate ureters; however, there was no ureteric stone or dilatation. Malrotation of both kidneys and moderate left hydronephrosis with multifocal parenchymal thinning could be seen, along with an 18-mm distal ureteric stone with an average density of 1250 Hounsfield unit (Figures 1, 2, and 3). The right-side crossed renal ectopia is supplied by two renal arteries. Both renal arteries arise below the level of the inferior mesenteric artery and are drained by multiple renal veins to the IVC (Figure 4). We counseled and consented the patient for cystoscopy, left retrograde pyelography (RGP), followed by ureteroscopy. Under spinal anesthesia, cystoscopy showed normal anterior and posterior urethra and left ureteric orifice. Two separate right ureteric orifices were identified. A guide wire was inserted up to the left kidney. RGP showed lower ureteric filling defect, moderate hydroureteronephrosis, and left duplex ureter, uniting proximally (Figure 5). The stone was seen impacted in the distal ureter. Stone fragmentation was carried out using a low-power holmium laser machine with a fiber size of 200 *μ*m (power: 1 J, frequency: 10 Hz, total energy delivered: 5.35 kJ). The lasing time was 16 min. All stone fragments were washed out from the ureter.

Finally, a 6-French/26-cm magnetic double-J stent and a 16-French Foley's catheter were inserted.

**Figure 6:**
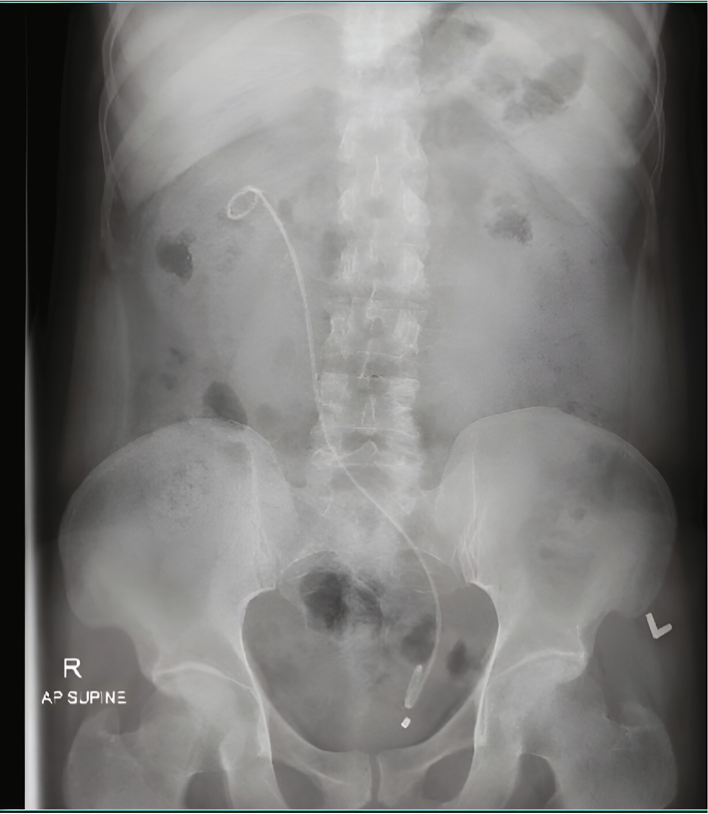
Postoperative x-ray of the abdomen showed a left double-J stent in place crossing to the right side.

After removing the urethral catheter, the patient was discharged home on the same day in a stable condition. Postoperative abdominal x-ray showed a left double-J stent in place with no evidence of residual stone (Figure 6). The stent was removed smoothly in the urology clinic after 3 weeks.

## 3. Discussion

CFRE is a very rare congenital malformation. The incidence is unknown because most patients are asymptomatic. It occurs in about 1 in 1000 live births [5]. Crossed renal ectopia is more prevalent in males than females, with a male-to-female ratio of 3:2. Left to right is more common than right to left. The arterial supply and the venous drainage of the CFRE are grossly abnormal [6, 7]. CFRE classification contains six types according to the degree of fusion, location, or rotation. These consist of (1) inferior crossed-fused ectopia, (2) sigmoid or S-shaped kidney, (3) unilateral lump kidney, (4) unilateral disc kidney, (5) L-shaped kidney, and (6) superior crossed-fused ectopia [8]. In our patient, the fusion was inferior crossed-fused ectopia.

Urological anomalies reported in conjunction with CFRE are ureteropelvic junction (UPJ) obstruction, vesicoureteral reflux, ectopic ureteroceles, and, rarely, multicystic dysplastic kidney [9]. The duplex system, either complete or incomplete, like in our case, is one of the rarest congenital anomalies associated with CFRE. In the available literature, no publication has found similar findings.

The fusion abnormalities are clinically significant, because half of the cases are complicated by obstruction, infections, and nephrolithiasis, like in our case. These complications occur because the abnormal kidney position and the anomalous blood supply may impede the urine drainage from the collecting system, creating a predisposition to urinary tract infection and calculus formation [10]. Some of these complications and their treatment might be challenging because of the abnormal, varying anatomy.

CFRE with a duplex-collecting system represents a unique and rare anatomical variant that requires meticulous evaluation and personalized management.

In general, techniques used to manage urinary tract calculi in CRFE include extracorporeal shock wave lithotripsy (ESWL), percutaneous nephrolithotomy (PCNL), laparoscopy, flexible uretero-nephoscopy, and open surgery in some complicated cases [4].

## 4. Conclusion

With our case report, we demonstrated that even in such complex urinary upper tract anomaly, described in the article, complicated with obstructing stone disease, endourological management is feasible and safe. Further documented cases would contribute to a deeper understanding of these conditions and would help to establish appropriate treatment strategies for affected individuals.

## Data Availability

The data that support the findings of this study are available from the corresponding author upon reasonable request.
